# Experimental severe malaria is resolved by targeting newly-identified monocyte subsets using immune-modifying particles combined with artesunate

**DOI:** 10.1038/s42003-018-0216-2

**Published:** 2018-12-13

**Authors:** Paula Niewold, Amy Cohen, Caryn van Vreden, Daniel R. Getts, Georges E. Grau, Nicholas J. C. King

**Affiliations:** 1 Viral Immunopathology, Discipline of Pathology and Bosch Institute, School of Medical Sciences, Faculty of Medicine and Health, University of Sydney, Camperdown, NSW 2050 Australia; 2 Vascular Immunology Unit, Discipline of Pathology and Bosch Institute, School of Medical Sciences, Faculty of Medicine and Health, University of Sydney, Camperdown, NSW 2050 Australia; 3 Sydney Cytometry, The University of Sydney and The Centenary Institute, Camperdown, NSW 2050 Australia; 4 Department of Microbiology-Immunology and Interdepartmental Immunobiology Center, Feinberg School of Medicine, Northwestern University, Chicago, IL 60611 USA; 5 TcR2, Therapeutics, 100 Binney Street, Suite 710, Cambridge, MA 02142 USA

**Keywords:** Innate immunity, Parasitic infection, Parasite host response, Translational immunology

## Abstract

Current treatment of severe malaria and associated cerebral malaria (CM) and respiratory distress syndromes are directed primarily at the parasite. Targeting the parasite has only partial efficacy in advanced infection, as neurological damage and respiratory distress are due to accumulation of host blood cells in the brain microvasculature and lung interstitium. Here, computational analysis identifies Ly6C^lo^ monocytes as a major component of the immune infiltrate in both organs in a preclinical mouse model. Specifically targeting Ly6C^lo^ monocyte precursors, identified by adoptive transfer, with immune-modifying particles (IMP) prevents experimental CM (ECM) in 50% of *Plasmodium berghei* ANKA-infected mice in early treatment protocols. Furthermore, treatment at onset of clinical ECM with 2 doses of a novel combination of IMP and anti-malarial drug artesunate results in 88% survival. This combination confers protection against ECM and mortality in late stage severe experimental malaria and provides a viable advance on current treatment regimens.

## Abstract (web summary)

Niewold, Cohen et al. identify Ly6C^lo^ monocytes as a major component of immune cell sequestration in a mouse model of severe malaria. They show that combined immune-modifying particles and anti-malarial drug treatment at the onset of disease signs results in 88% survival of malaria-infected mice.

## Introduction

Severe malaria is almost exclusively caused by the mosquito-borne parasite *Plasmodium falciparum*. Recent WHO estimates indicate 212 million cases of malaria worldwide in 2015, with 429,000 individuals dying from severe malaria^1^. Severe malaria comprises a constellation of serious complications, including cerebral malaria (CM), respiratory distress (RD), severe malarial anaemia and metabolic abnormalities. CM is associated with cerebral blood vessel occlusion by adherent, parasitised red blood cells (pRBC), platelets and leucocytes. This results in focal areas of brain ischaemia that progress to blood–brain barrier breakdown and vascular leakage, causing delirium, seizures and coma in patients^2–4^. Similar vascular involvement in the lungs results in blood vessel leakage and interstitial leucocyte accumulation, leading to malaria-associated RD and acute respiratory decompensation^5^. Both syndromes are in most cases lethal without treatment.

Current malaria treatments almost exclusively target the parasite. The WHO-recommended treatment for severe malaria is parenteral artesunate for 24 h, followed by 2 days of artemisinin-based combination therapy^6,7^. However, overall mortality on this regime remains 10–20% and is projected to increase with the spread of artemisinin-resistant parasites^8,9^. Adjunct immunomodulatory treatment approaches in RD and CM syndromes do not specifically target causative cellular mediators and to date have not been successful in ameliorating disease^10^.

Intravascular sequestration of monocytes has been identified in both human CM^2,3,11^ and mouse experimental CM (ECM)^12^ and these cells are a major source of pro-inflammatory cytokines and chemokines in CM pathogenesis. The pathological involvement of these cells is emphasised by the abrogation of the syndrome in *Plasmodium berghei*-ANKA (PbA)-infected mice after global monocyte/macrophage depletion by clodronate liposomes^13^ or reduced inflammatory monocyte recruitment by anti-CCR2 antibody^14^. CCR2−knockout mice have reduced monocyte accumulation and pathology in a murine model of RD^15^. However, these approaches are only effective at or prior to the time of inoculation, which is an unrealistic timeframe for clinical application.

We have previously demonstrated that immune-modifying particles (IMP) selectively modulate circulating inflammatory Ly6C^hi^ monocytes in a variety of diseases without impeding the development of a robust immune response^16^. Monocytes phagocytosing IMP in the bloodstream are sequestered in the spleen and thus prevented from migrating to inflammatory foci. In a murine model of West Nile virus encephalitis, in which the accumulation of Ly6C^hi^ monocytes in the brain is uniformly lethal, IMP treatment was more effective than clodronate or anti-CCL2 antibody treatment^17^. Importantly, abrogation of encephalitis was achieved when IMP were administered after development of clinical signs^16^.

Here, we use computational analysis to separate Ly6C^lo^ monocytes from microglia and identify them as a novel major subset associated with pathology in both the brain vasculature and lungs in severe malaria. The importance of monocytes in severe malaria is supported by the efficacy of IMP treatment in early infection in a lethal preclinical murine ECM model. The use of a directed immune-modulatory approach in severe malaria is novel, and combined with anti-parasitic artesunate, rescues nearly 90% of mice presenting with neurological symptoms, as well as abrogating RD in the lung.

## Results

### Ly6C^lo^ monocytes are the main subset in vasculature during ECM

**Fig. 1:**
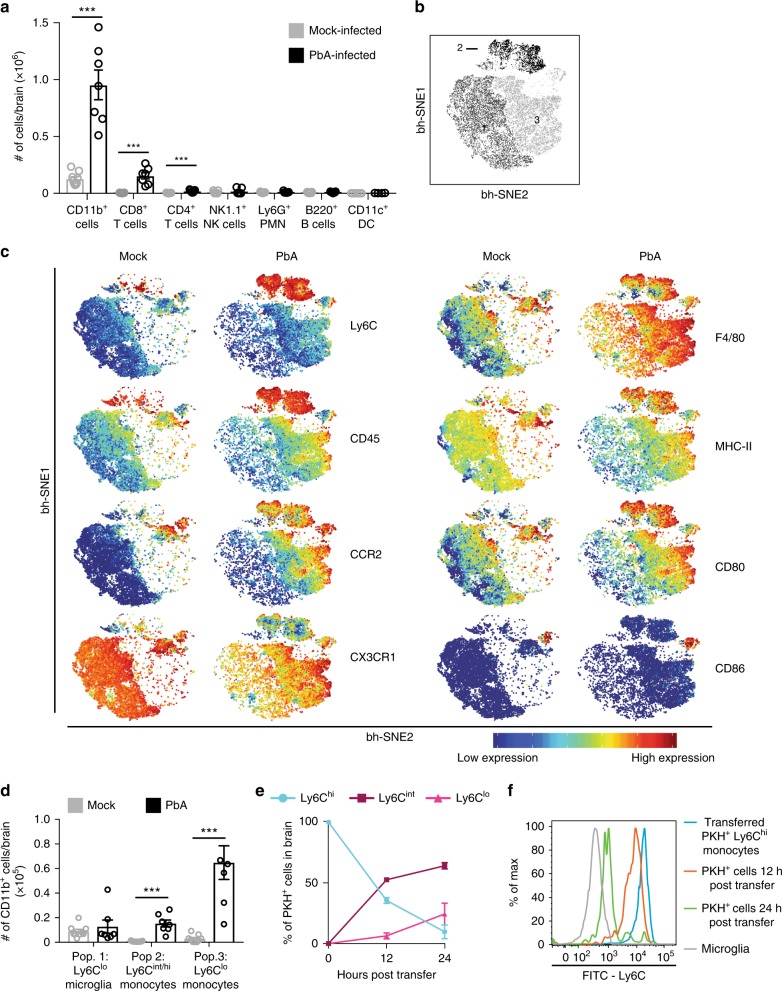
The major infiltrating Ly6C^lo^ population in the brain is derived from Ly6C^hi^ monocytes. **a** Flow cytometry was used to determine the proportional size of specific populations in the brain of mock- (grey) and PbA-infected (black) mice on d8 p.i. Numbers were determined by multiplying organ cell counts with the percentages obtained in FlowJo. **b** Representative visual ‘legend’ of populations formed by tSNE analysis on the CD11b^+^ population of flow cytometry data of the brain of mock- and PbA-infected mice on d8 p.i. **c** Representative heat-maps of relative marker expression on the CD11b^+^ population in the brains of mock- and PbA-infected mice. **d** Numerical representation of CD11b^+^ populations in the brain of mock- and PbA-infected mice identified by tSNE, gated in FlowJo. **e** CD11b^+^Ly6G^−^Ly6C^hi^ inflammatory monocytes were isolated from the bone marrow of PbA-infected mouse donors on d7 p.i., labelled with PKH26 fluorescent dye, and injected into matched recipients on d7 p.i. Brains of recipient mice were processed 12 or 24 h later, on d8 p.i. and adoptively-transferred PKH26^+^ cells were identified and their Ly6C expression determined (blue: Ly6C^hi^, maroon: Ly6C^int^, pink: Ly6C^lo^). **f** Representative histograms of Ly6C expression on microglia (grey) and on PKH^+^ populations in the brain at 0 (blue), 12 (orange) and 24 (green) hours post-transfer. Data in **a** and **d** represent three separate experiments with a total no. of 7–9 mice per group, shown as mean ± SEM, and analysed using a Mann–Whitney test. Data in **e** represents two independent experiments with a total no. of 3–6 mice per group, shown as mean ± SEM. Source data are provided as a Source Data file

CM develops as a result of accumulating parasitised RBC, platelets and leucocytes causing vascular occlusion in the brain^3^. We investigated this immune-mediated pathology in a preclinical mouse model of ECM, which reproduces more than 25 clinical, biochemical, pathophysiological, histopathological and immunological features of human disease^18–21^. The brains of mice at the time of clinical ECM on d8 after PbA inoculation, with a mean clinical score of 3.4, were analysed by flow cytometry. This showed that CD11b^+^ cells constituted ~80% of accumulating leucocytes in the brain vasculature, while T cells, B cells, neutrophils, dendritic cells and natural killer cells were numerically minor populations (Fig. 1a) (gating shown in Supplementary Fig. 1). Clustering analysis using t-distributed stochastic neighbour embedding (tSNE) was applied to further characterise the numerically major CD11b^+^ population in the brain. This analysis creates a two-dimensional figure in which the position of each cell is determined by its relation to other cells, based on the expression of fluorescence and scatter markers in multi-dimensional flow cytometric analysis^22^. This results in the discrete grouping of cells with similar expression patterns. Analysis of the CD11b^+^ population from the brains of mock-infected and PbA-infected mice on d8 p.i. by tSNE consistently showed three distinct subpopulations (Fig. 1b), while manual gating only resolved two (Supplementary Fig. 1A). To characterise these three populations, their expression of relevant markers was compared by representing the tSNE output as an expression heat map (Fig. 1c). Low expression of Ly6C, CD45 and CCR2, and high expression of CX3CR1, as observed on population 1, is consistent with a microglial phenotype, which is most prominent in mock-infected animals^23,24^. In contrast, cells in population 2 expressed high levels of Ly6C, CD45 and CCR2, and low levels of CX3CR1, consistent with a BM-derived inflammatory monocyte phenotype more obvious in PbA-infected mice^24,25^. Population 3 expressed low levels of Ly6C and high levels of CX3CR1, with intermediate expression of CCR2 and CD45 and was also more prominent in PbA-infected mice. Although Ly6C^lo^ and CX3CR1^hi^ expression is observed on microglia, this pattern is also found on Ly6C^lo^ monocytes, and CX3CR1 expression on the latter is associated with ligand-mediated arrest on activated vascular endothelium^26,27^. The intermediate expression of both CD45, the pan-leucocyte marker, and CCR2, which is involved in BM egress, clearly separate these Ly6C^lo^ cells from the microglial population and strongly suggest they are BM-derived rather than brain-resident cells. The expression of MHC-II and CD80 on populations 2 and 3 support a monocyte/macrophage phenotype and further distinguish them from the microglia in population 1, which exhibit lower expression of these markers. F4/80 expression, in combination with the above markers on populations 2 and 3 is consistent with a macrophage phenotype, but F4/80 expression has also been shown on microglia^28^. Thus, microglia (population 1) and Ly6C^lo^ monocytes (population 3) are indistinguishable based on Ly6C, CX3CR1 or F4/80 expression (Fig. 1c and Supplementary Fig. 2), and minor differences in CD45 and CD11b expression are insufficient to separate these populations. Even the larger difference in CCR2 expression is insufficient to separate the populations fully on histogram or 2D plots. However, the inclusion of all markers in tSNE analysis allowed us to identify non-resident Ly6C^lo^ monocytes as the most prevalent population in the ECM brain vasculature. To our knowledge, this is the first time this distinction has been shown in malaria.

Analysis by tSNE also reveals a small cluster of CD86^+^ cells between populations 2 and 3, that expresses slightly more CD45, Ly6C, CCR2, but less CX3CR1, than Ly6C^lo^ monocytes (population 3), and may represent monocytes in transition from Ly6C^hi^ to Ly6C^lo^.

In mock-infected mice, the brain was largely dominated by microglia (population 1), with small numbers of Ly6C^hi^ and Ly6C^lo^ monocytes (populations 2 and 3, Fig. 1d). In PbA-infected mice, Ly6C^lo^ monocytes were by far the largest population, increasing to seven-fold that of microglia, while Ly6C^hi^ monocytes had also increased significantly, compared to the mock-infected controls. We hypothesised that CD45^int^ CCR2^int^ Ly6C^lo^ monocytes (population 3) accumulating in the brain vasculature were derived from CD45^hi^ CCR2^hi^ Ly6C^hi^ inflammatory monocytes (population 2). The latter are recruited to sites of inflammation from the BM and can differentiate into Ly6C^lo^ monocytes in both humans and mice^29,30^. To investigate this in our model, Ly6C^hi^ monocytes were sorted from the BM of d8 p.i. PbA-infected mice (gating strategy shown in Supplementary Fig. 3), labelled with PKH26 and injected into a separate group of PbA-infected mice 12 or 24 h prior to endpoint disease. After 12 h, > 50% of PKH^+^ cells in the brain had acquired a Ly6C^int^ or Ly6C^lo^ profile and by 24 h only 10% of transferred cells remained Ly6C^hi^ (Fig. 1e). The slopes of the graphs indicate that there is a transition of expression from Ly6C^hi^ to Ly6C^int^ cells in the first 12 h. This slows between 12 and 24 h, when increasing numbers of transferred cells acquire a Ly6C^lo^ phenotype. This is reflected in the sharp decrease in Ly6C expression seen on the transferred cells by 24 h, which approach the levels observed on microglia (Fig. 1f).

### Interstitial macrophages and Ly6C^lo^ monocytes accumulate in RD

**Fig. 2:**
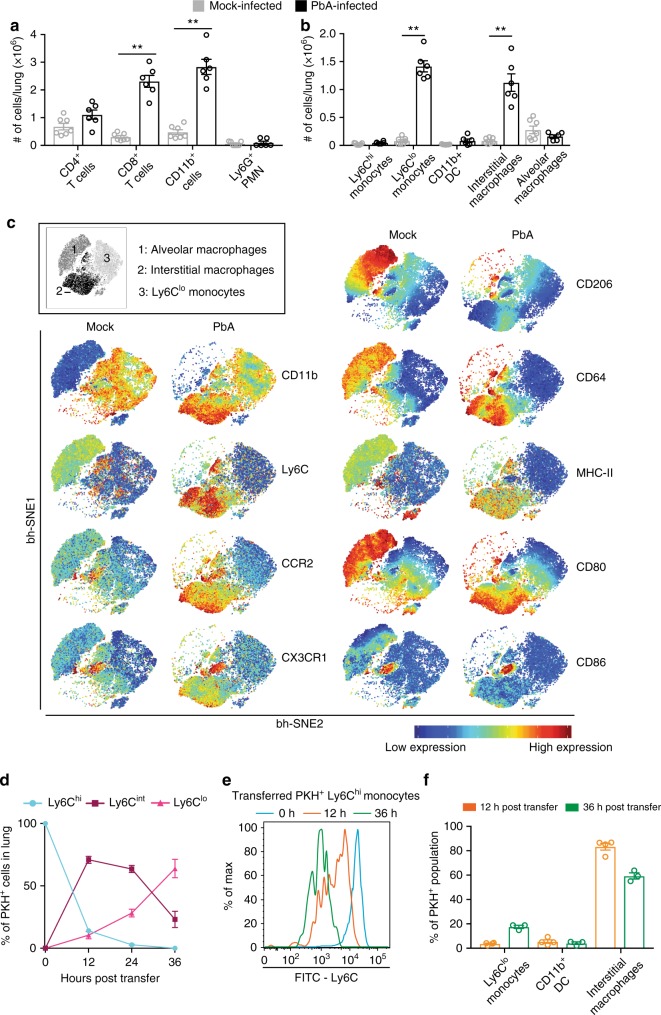
Major infiltrating cell populations in the lung are derived from Ly6C^hi^ monocytes. **a**, **b** Flow cytometry was used to determine the proportional size of specific populations in the lungs of mock- (grey) and PbA-infected (black) mice on d8 p.i. Numbers were determined by multiplying organ cell counts with the percentages obtained in FlowJo. **c** Representative visual ‘legend’ of populations formed by tSNE analysis on the CD11b^+^ population in flow cytometry data of the lungs of mock- and PbA-infected mice on d8 p.i. (inset) and representative heat-maps of relative marker expression on these populations. **d** CD11b^+^Ly6G^−^Ly6C^hi^ inflammatory monocytes were isolated from the bone marrow of PbA-infected mouse donors on d7 p.i., labelled with PKH26 fluorescent dye, and injected into matched recipients on d6 or d7 p.i. Lungs of recipient mice were processed 12, 24 or 36 h later, on d8 p.i. and adoptively transferred PKH26^+^ cells were identified and their Ly6C expression determined (blue: Ly6C^hi^, maroon: Ly6C^int^, pink: Ly6C^lo^). **e** Representative histograms of Ly6C expression on PKH^+^ populations in the lung at 0 (blue), 12 (orange) and 36 (green) hours post-transfer. **f** Flow cytometric identification of PKH^+^ bone marrow-derived CD11b^+^Ly6G^−^Ly6C^hi^ monocytes found in the lung 12 and 36 h post-transfer. Data in **a** and **b** represent two separate experiments with a total no. of 6–7 mice per group, shown as mean ± SEM, and analysed using a Mann–Whitney test. Data in **d** and **f** represent one experiment with 3–4 mice per group, shown as mean ± SEM.  Source data are provided as a Source Data file

RD is a serious complication of severe malaria caused by pulmonary leucocyte accumulation^5^. In contrast to the brain, where cells are confined to the vascular lumen, in the lung they also extravasate into the interstitium^31,32^. Histologically, the majority of these cells have been identified as macrophage-like in both mice and humans^32^, however, more detailed subset analysis is lacking. Increased levels of protein in the lung are indicative of RD and found from d7 p.i. onwards^33^. Flow cytometric analysis on d8 p.i. confirmed the CD11b^+^ population makes up a large proportion of accumulating cells in the lung, although T cells also constitute a significant part of the infiltrate (Fig. 2a). We characterised the CD11b^+^ population, including alveolar macrophages, using a gating strategy previously described in a murine lung fibrosis model^34^ (Supplementary Fig. 4). We observed a significant increase in CD11b^+^ cell numbers, especially Ly6C^lo^ monocytes (18-fold) and interstitial macrophages (14-fold) in PbA-infected mice, compared to mock-infected controls, while numbers of tissue-resident alveolar macrophages did not change significantly (Fig. 2b). These three populations were clearly distinguishable on tSNE analysis (Fig. 2c, inset). Alveolar and interstitial macrophages (populations 1 and 2, respectively) had a similar expression pattern of CD64, MHC-II, CD80 and CD86, indicating a macrophage phenotype. The higher expression of CCR2 and CD11b on interstitial macrophages suggests they originate from bone marrow, while alveolar macrophages have low expression of these markers and high expression of CD206, consistent with a tissue-resident phenotype^35^ (Fig. 2c). Ly6C^lo^ monocytes (population 3) are CD11b^+^, indicating their myeloid origin, but they have relatively low expression of the other markers measured here (Fig. 2c). Similar to the brain, a small CD86^+^ cluster located between population 2 and 3 may represent transitioning cells.

Since Ly6C^lo^ monocytes and interstitial macrophages are associated with acute inflammation in the lung^34^, we hypothesised they developed from BM-derived Ly6C^hi^ monocytes, similar to the Ly6C^lo^ monocytes in the ECM brain. Similar to the brain, Ly6C expression on adoptively transferred Ly6C^hi^ monocytes was markedly reduced within 12 h, with ~70% of PKH^+^ cells identified as Ly6C^int^ and ~10% as Ly6C^lo^ (Fig. 2d). At 24 h, the proportions of Ly6C^int^ and Ly6C^hi^ cells were reduced, in favour of an increase in Ly6C^lo^ cells. By 36 h, none of the transferred cells in the lung remained Ly6C^hi^ and > 60% had become Ly6C^lo^, while Ly6C^int^ cell proportions were further reduced. Accordingly, histograms of the PKH^+^ populations show a reduction of Ly6C expression comparable to that in the brain (Fig. 2e). Further analysis of the PKH^+^ population revealed that by 12 h post-transfer ~80% of cells were identifiable as interstitial macrophages. This proportion was reduced by 36 h post-transfer, while Ly6C^lo^ monocytes had increased (Fig. 2f). Thus, the differentiation of Ly6C^hi^ monocytes to Ly6C^int^ and Ly6C^lo^ subsets mirrors that in the brain vasculature.

### IMP reduce monocyte infiltration and increase survival in ECM

**Fig. 3:**
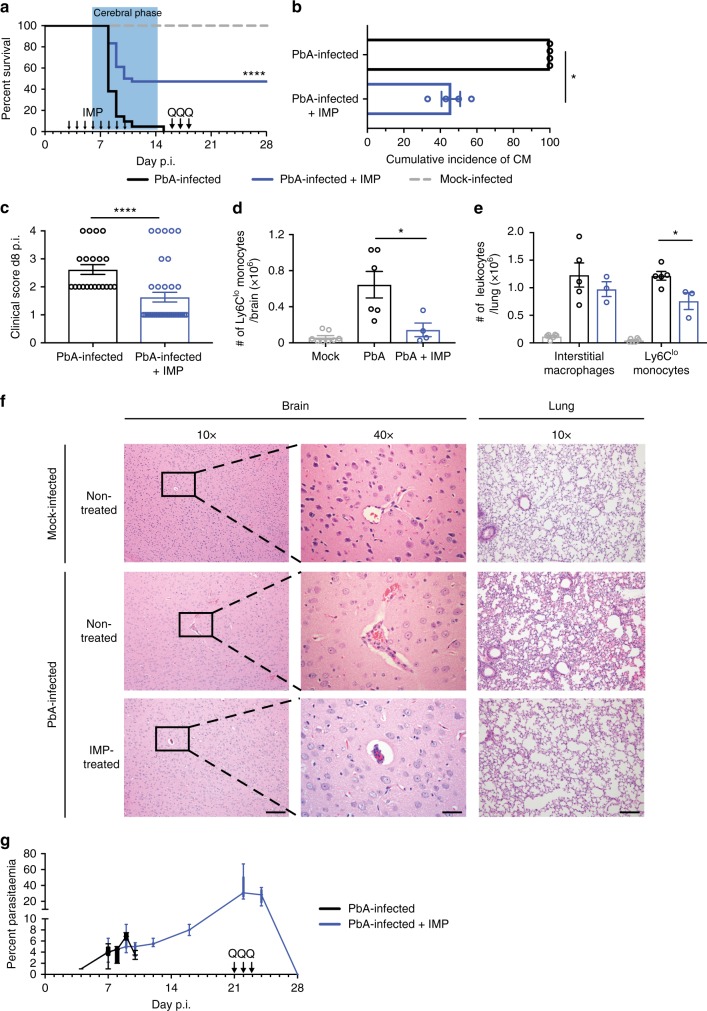
IMP treatment increases survival of PbA-infected mice. **a** Survival of mice over time post PbA infection, comparing IMP treatment from d3–10 p.i. (blue) with untreated controls (black), and mock-infected mice (grey). The acute phase of mortality, observed during the second week post PbA infection, is denoted by the blue box, and the arrows annotate initiation of IMP and each of three quinine treatments. **b** Cumulative incidence of the neurological syndrome between d6 and d14 p.i. in PbA-infected mice, with or without IMP treatment. **c** Average clinical score on d8 p.i. in PbA-infected mice receiving different treatments, shown as mean ± SEM. **d** Number of Ly6C^lo^ monocytes in the brain of mock-infected, PbA-infected untreated and PbA-infected IMP-treated (d3–d7) mice on d8 p.i. **e** Number of interstitial macrophages and Ly6C^lo^ monocytes in the lung of mock-infected, PbA-infected untreated and PbA-infected IMP-treated (d3–d7) mice on d8 p.i. **f** Representative haematoxylin and eosin-stained brain and lung sections from mock- or PbA-infected mice with or without IMP treatment on d8–10 p.i. Scale bars are equal to 100 or 20 μm for 10x and 40x images, respectively. **g** Peripheral blood parasitaemia levels in treated and untreated infected mice. Data represents four separate experiments with a total no. of 21–36 mice per group for **a**–**c** and **g**. Data in **a** was compared using the Mantel–Cox log-rank test. **d** and **e** show data representing two separate experiments with a total no. of 5–6 per group. Data in **b**–**e** are shown as mean ± SEM and were analysed using a Mann–Whitney test. IMP and Q indicate the timepoints at which IMP and quinine treatment were given.  Source data are provided as a Source Data file

To reduce the major accumulating monocyte populations in the brain vasculature and lung during severe malaria, we targeted precursor Ly6C^hi^ monocytes in the bloodstream, prior to their arrival at sites of inflammation. We previously showed that uptake of immune-modifying particles (IMP) by circulating Ly6C^hi^ monocytes results in their sequestration in the spleen, thereby reducing their accumulation at inflammatory sites^16^. Therefore, we administered IMP intravenously (i.v.) into PbA-infected mice daily for 7 days from d3 p.i., when monocyte sequestration is first detectable in the brain vasculature. IMP treatment resulted in 48% survival, while untreated mice uniformly succumbed to ECM (Fig. 3a). Surviving IMP-treated mice did not develop clinical signs of ECM, as reflected in the lower cumulative incidence of ECM, and the significantly lower average clinical score on d8 p.i., compared to untreated controls (Fig. 3b, c, respectively). Surviving IMP-treated mice were given three daily doses of quinine on d16–18 p.i. to abrogate the development of anaemia following the cerebral phase (Fig. 3a, arrows). Increased survival in IMP-treated, PbA-infected mice was associated with a dramatic reduction in numbers of Ly6C^lo^ monocytes in the brain, with treatment reducing numbers almost to mock-levels (Fig. 3d). In the lung, numbers of Ly6C^lo^ monocytes were also significantly reduced, with a smaller reduction in the numbers of interstitial macrophages (Fig. 3e). This was also associated with the accumulation of Ly6C^hi^ monocytes in the spleen in IMP-treated animals (Supplementary Fig. 5). Histology of the brain vasculature showed that sequestration of RBC and leucocytes, invariably seen in PbA-infected mice, was markedly reduced in PbA-infected, IMP-treated mice (Fig. 3f). In the lungs of these mice, there was a marked reduction in the typical alveolar septal thickening associated with cellular infiltration (Fig. 3f). IMP treatment did not result in reduced parasitaemia (Fig. 3g). However, quinine treatment effectively cleared the parasite and IMP-treated mice subsequently survived for more than a year.

### IMP and artesunate synergise to ameliorate overt ECM and RD

**Fig. 4:**
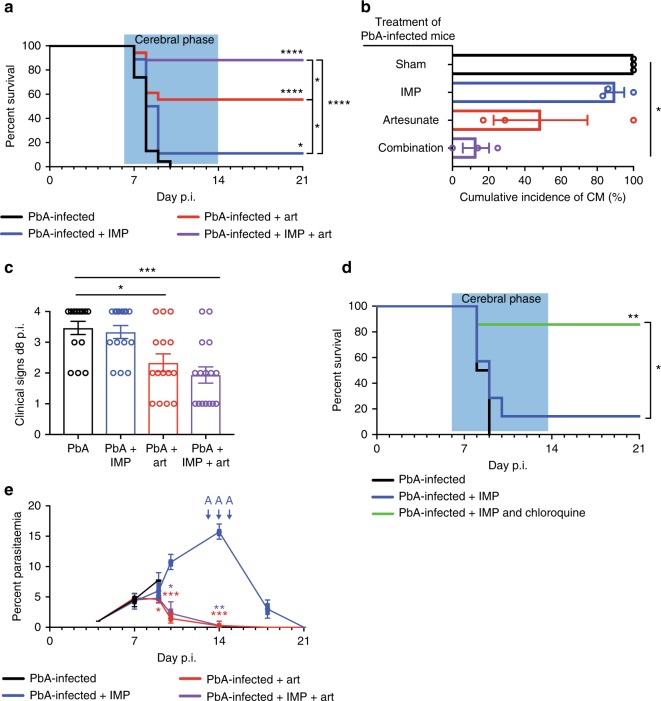
IMP and artesunate combination treatment upon ECM presentation increases survival. **a** Survival of mice over time post PbA infection, comparing IMP (blue), artesunate (red) or combination treatment (purple) from onset of ECM symptoms with untreated controls (black). The acute phase of mortality, observed during the second week post PbA infection, is denoted by the blue box. **b** Cumulative incidence of the neurological syndrome between d6 and d14 p.i. in PbA-infected mice receiving different treatments. **c** Average clinical score on d8 p.i. in PbA-infected mice receiving different treatments, shown as mean ± SEM. **d** Survival of mice over time post PbA infection, comparing treatment from onset of ECM symptoms with IMP alone (blue) or in combination with chloroquine (green), with untreated mice. **e** Peripheral blood parasitaemia levels in PbA-infected mice treated with IMP, artesunate or a combination treatment from d7 p.i. and untreated controls. Data in **a**–**c** and **e** represents three separate experiments with a total no. of 17–23 mice per group. Data in **d** represents three separate experiments with a total no. of 5–11 mice per group. Data in **a** and **d** were compared using the Mantel–Cox log-rank test. Data in **c** is shown as mean ± SEM and were analysed using a Kruskal–Wallis test with a Dunn’s multiple comparison test and PbA as the control group. Data in **e** is shown as mean ± SEM and were analysed using a Kruskal–Wallis test with a Dunn’s multiple comparison test comparing all groups at each timepoint.  Source data are provided as a Source Data file

While the above confirms that inflammatory monocytes are a viable target in PbA infection, to apply this in a clinically relevant timeframe, we initiated treatment at the onset of clinical signs of disease. Since anti-parasite drug treatment was required following IMP-mediated survival, IMP treatment was combined with the anti-malarial drug, artesunate, as recommended for severe malaria by the WHO^7^. At the onset of ECM signs, as indicated by a clinical score of 2, between d6 and 8 p.i., a novel combination of artesunate and IMP was administered, with a second dose 24 h later. To ensure each treatment was initiated at the same stage of disease and minimise possible treatment bias, mice were allocated randomly and progressively to treatment groups as they exhibited ECM signs between d6 and d8 p.i. Combination treatment resulted in a survival rate of 88%, while artesunate or IMP treatment alone resulted in 56% and 10% survival, respectively (Fig. 4a).

The synergistic effect of combining artesunate and IMP treatment was evident in the lower cumulative incidence score and the reduced average clinical score on d8 p.i. (Fig. 4b, c). Interestingly, treatment combining IMP and chloroquine—an alternative anti-malarial therapy—also had a strong protective effect against ECM (Fig. 4d), despite the lower efficacy of chloroquine treatment alone, compared to artesunate, in both mice and humans^36,37^. As expected, mice treated with artesunate, alone or in combination with IMP, were able to clear the parasite, while mice treated only with IMP required three doses of artesunate to prevent anaemia and achieve long-term survival (Fig. 4e).

Cerebral pathology in mice and humans is also associated with increased numbers of MV, which are linked to increased cellular adhesion^38,39^. Blocking their release protects against CM^40^. Since monocytes are one of the main sources of MV during CM^41^, we investigated the effect of treatment on the number of plasma MV in these groups. Circulating MV numbers were significantly decreased in mice treated with IMP, either alone or in combination with artesunate, but not in mice treated with artesunate alone (Supplementary Fig. 6).

**Fig. 5:**
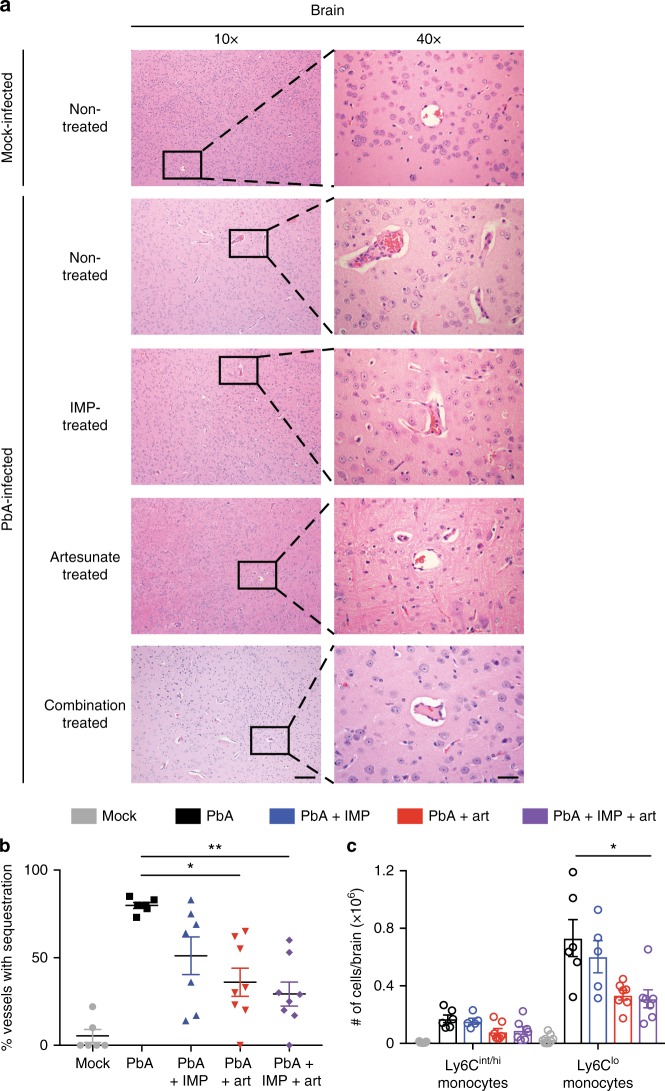
IMP and artesunate treatments result in improved pathology in the brain of PbA-infected mice. **a** Representative haematoxylin and eosin brain sections from mock mice, and PbA-infected mice with no treatment, or treatment with IMP, artesunate or a combination of the two, on d8–10 p.i. Scale bars are equal to 100 or 20 μm for 10x and 40x images, respectively. **b** Occluded vessels in entire brain sections were enumerated as previously described in ref. ^62^. The proportion of capillaries containing sequestered leucocytes or RBC compared to empty vessels was calculated in PbA infection, comparing IMP (blue), artesunate (red) or combination treatment (purple) from onset of ECM symptoms with untreated controls (black) and mock-infected (grey) animals. **c** Flow cytometry was used to gated and enumerate specific CD11b^+^ populations in the brain 36 h after initial treatment. Data in **b** and **c** represent three separate experiments with a total no. of 6–9 mice per group, shown as mean ± SEM, and analysis was performed using a Kruskal–Wallis test with a Dunn’s multiple comparison test and PbA as the control group.  Source data are provided as a Source Data file

**Fig. 6:**
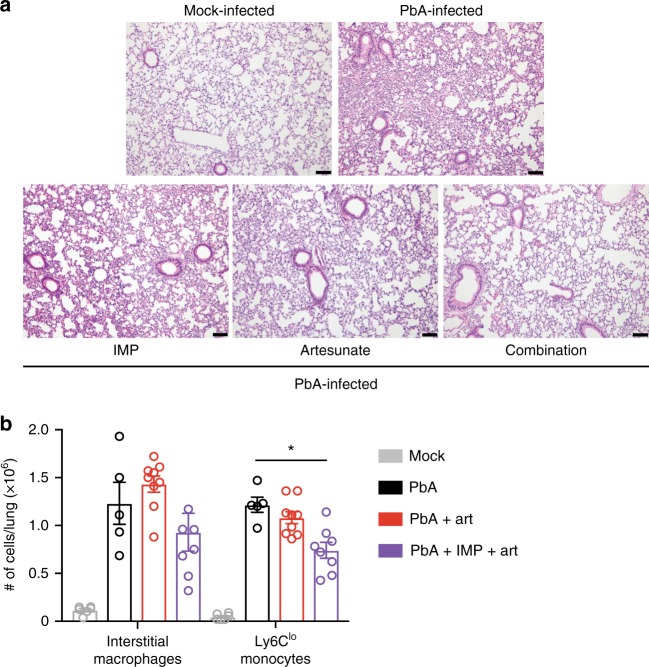
IMP and artesunate treatments result in improved pathology in the lung of PbA-infected mice. **a** Representative haematoxylin and eosin lung sections from mock mice, and PbA-infected mice with no treatment, or treatment with IMP, artesunate or a combination of the two, on d8–10 p.i. Images were taken at 10x and scale bars are equal to 100 μm. **b** Flow cytometry was used to gate and enumerate specific CD11b^+^ populations in the lung 36 h after initial treatment in PbA infection, comparing artesunate (red) or combination treatment (purple) from onset of ECM symptoms with untreated controls (black) and mock-infected (grey) animals. Data in **b** represents two experiments with a total *n* of 3–7 mice per group, shown as mean ± SEM, and were analysed using a Kruskal–Wallis test with a Dunn’s multiple comparison test and PbA as the control group.  Source data are provided as a Source Data file

Histological examination of mice treated with artesunate and IMP in combination showed no evidence of cerebral haemorrhage and very little vascular cell sequestration on d8 p.i., in sharp contrast to the untreated controls (Fig. 5a). The few nucleated cells visible in the vessels of mock-infected and treated mice were endothelial cells, as indicated by their flattened nuclei, in contrast to the sequestered leucocytes visible in infected, untreated mice. Artesunate-treated mice also showed substantially reduced vascular occlusion, while there was less improvement in mice treated with IMP alone. Enumeration of the occluded vessels in the brains showed reduced occlusion in all treatment groups. This was statistically significant in the artesunate alone and combination treatment groups, but more pronounced in the latter (Fig. 5b). Consonant with this, flow cytometry showed vascular leucocyte accumulation was most reduced following combined artesunate and IMP treatment; numbers of Ly6C^hi/int^ monocytes dropped from 1.70 × 10^5^ to 0.68 × 10^5^, while, strikingly, Ly6C^lo^ monocytes were reduced by almost 5 × 10^5^, i.e*.*, from 7.3 × 10^5^ to 2.5 × 10^5^ (Fig. 5c). In the lungs, combination treatment was also most effective, producing the greatest reduction in interstitial thickening and cellular infiltrate, while separately, IMP or artesunate treatment alone reduced pathology somewhat (Fig. 6a). Artesunate treatment had no significant effect on the numbers of interstitial macrophages and Ly6C^lo^ monocytes in the lung (Fig. 6b). Combination treatment, however, reduced numbers by ~50%. Treatment with IMP alone was excluded from this analysis, as survival numbers were low in this group. These findings correspond with the improvement in clinical signs in combination-treated mice and emphasise the pathological nature of monocyte accumulation in severe malaria.

Since these treatments abrogated ECM and afforded long-term survival, we rechallenged mice to assess their immunity to PbA. Remarkably, reinfection of survivors from both early treatment protocols (Fig. 3a) resulted in minimal clinical signs and parasitaemia and complete survival, compared to naïve PbA-infected mice (Supplementary Fig. 7A–C). Furthermore, mice surviving primary infection following late treatment with IMP, artesunate or a combination of both, all survived lethal dose rechallenge (Supplementary Fig. 7D). Overall, the addition of targeted immune-modulating treatment almost doubles the efficacy of existing anti-parasitic interventions in late stage disease.

## Discussion

Treatment of severe malaria is generally aimed at clearing the parasite, with artemisinin-based combination therapy the current recommended treatment approach^7^. Notwithstanding, there is significant patient mortality, especially in individuals treated late in clinical presentation^6^, and this is likely to rise with increasing parasite resistance^8,9^. While the presence of the parasite is clearly required for development of the syndrome, the dysregulated immune response crucially contributes to vascular occlusion in the brain and major leucocyte accumulation in the lung, resulting in lethality. We characterised the immune cell infiltrate to identify potential targets for immune-modulating therapy. Previously, accumulations of macrophage-like cells in the brain and lungs in severe malaria have been observed principally by histology. Here, cluster analysis of detailed phenotypic data specifically identifies Ly6C^lo^ monocytes as the major population in the brain at peak disease, and clearly distinguishes them from microglia. Importantly, in the lung, cluster analysis also identified Ly6C^lo^ monocytes as the major myeloid population, while interstitial macrophages were a separate substantial population. Our data suggest these cells are derived from Ly6C^hi^ inflammatory monocytes originating in the bone marrow. Indeed, specifically targeting these cells with IMP markedly reduced monocyte accumulation in both organs and increased long-term survival with immunity to lethal rechallenge. Notably, administration of IMP in combination with anti-malarial compounds at the onset of clinical signs of severe malaria rescued almost 90% of PbA-infected mice, regardless of whether artesunate or chloroquine was used. Thus, we show that the combination of specific immune system-targeted and anti-parasitic therapy provides substantial advantage over either of these approaches alone. This also has potentially important implications for ongoing success of treatment in the face of increasing drug resistance of the malaria parasite.

An increase in CD11b^+^ cells in the brain has previously been observed in CM, however, the phenotype(s) of these cells have not been defined in detail. It has been suggested that CM results in significant microglial proliferation, with pathogenic involvement of these cells, which were identified as CX3CR1^+^ and Ly6C^lo^^42^. The additional parameters used in our study and analysed by tSNE enabled us to distinguish microglia from a separate population of Ly6C^lo^ monocytes, despite their similar expression of CX3CR1 and Ly6C. This analysis reveals that microglial numbers do not change in ECM, while Ly6C^lo^ monocytes accumulate intravascularly in large numbers. Previously, the accurate distinction between microglia and infiltrating Ly6C^lo^ monocytes has relied on the use of reporter mice with fluorescent protein expression in BM-derived cells^43^. Recently, Tmem119 was identified as a novel marker uniquely expressed on microglia in mice and humans^44^, but in our hands did not give microglia-specific staining in this model of malaria.

We further showed that bone marrow-derived Ly6C^hi^ monocytes become Ly6C^lo^ monocytes in the brain vasculature. This is consistent with previous work showing Ly6C^hi^-to-Ly6C^lo^ differentiation of monocytes in mice^30,45^ and equivalent populations in humans^29^. The accumulation of CX3CR1^+^Ly6C^lo^ monocytes in ECM is likely mediated by binding to endothelial fractalkine, which is upregulated in response to TNF in severe malaria^27,46,47^. The reduced Ly6C and increased CX3CR1 expression on these cells aligns with previous work showing that monocytes accumulate in the vasculature of the brain and acquire tissue macrophage features without transmigrating across the blood–brain barrier^12^. In malaria-infected individuals the numbers of CD14^lo^CD16^+^ monocytes, the human equivalent of this subset, have also been found to be increased. These cells are CCR2^-^ and express high levels of CX3CR1, which is correlated with increased fractalkine-mediated adherence to endothelial cells in vitro^48,49^.

In the lung, our results show that Ly6C^hi^ monocytes differentiate principally into Ly6C^int^ interstitial macrophages and Ly6C^lo^ monocytes. Our analysis and adoptive transfer data indicate interstitial macrophages are an intermediate stage between Ly6C^hi^ and Ly6C^lo^ monocytes in infected mice. This is consonant with previous work in a model of lung fibrosis which showed that infiltrating CD11b^+^ macrophages are derived from Ly6C^hi^ monocytes and CCR2 expression is required for their accumulation in the lung^50^. In PbA infection of C57BL/6 mice, CCR2 is also required for macrophage-derived monocyte accumulation in the lung^15^. The association between levels of CCL2 in the CSF and serum, and severity of disease in CM patients further supports the importance of CCR2^+^ cells (or their derivatives) in CM^51^.

Accumulation of adherent leucocytes, including monocytes, in brain vessels is limited in the early stages of infection, but increases significantly in mice displaying severe clinical signs^13^. Thus, early IMP treatment from d3 p.i. largely prevented intravascular monocyte accumulation and protected approximately half of the mice against ECM. However, at later timepoints when most monocytes were already present in the brain vasculature and had downregulated Ly6C expression, the efficacy of IMP treatment when administered alone was reduced. Consistent with this, in other studies, anti-CCR2 antibody treatment, which also targets Ly6C^hi^ monocytes, was successful early in infection, but not at later timepoints^14^.

Solely treating with anti-malarial drugs on clinical presentation of severe malaria also has limited efficacy in mice, with few published examples of successful treatment after the development of disease signs. In particular, RD can develop before, during or after administration of anti-parasitic treatment and mortality ranges between 50 and 80%, depending on the availability of mechanical ventilation^52^. This is consistent with the limited efficacy of artesunate in treating lung pathology observed in this study (Fig. 6a). In ECM, administration of artesunate or artemether at clinical disease onset resulted in 45% survival and reduced leucocyte accumulation in the brain vasculature, similar to our findings (Fig. 4a) and^53^. In other studies, twice daily treatment with artesunate, artemisone, chloroquine or a combination of these, between d6 and d9 p.i. prevented or delayed development of ECM, but required additional anti-parasitic treatment upon recrudescence^36,54^. Thus, in these studies treatment was required over an extended period and at least ten doses were needed to achieve long-term survival.

In contrast, the combination of artesunate and IMP, administered at the onset of neurological signs, effectively abrogated ECM and RD with only two doses over 48 h. This was associated with reduced pathogenic accumulation of monocytes in the cerebral vasculature and lungs. IMP-mediated reduction in circulating Ly6C^hi^ monocyte numbers would synergise with artesunate-mediated reduction in TNF levels, which itself would reduce endothelial ICAM-1 expression and thus leucocyte adhesion^45,55^. In the ECM brain, it seems that the reduction in Ly6C^lo^ monocytes in combination-treated mice may principally be attributable to the anti-adhesive activity of artesunate, with IMP contributing to the reduction in circulating Ly6C^hi^ monocytes. In the lung, the effect of combination treatment may be due more to the action of IMP reducing circulating Ly6C^hi^ monocyte numbers, since in the lung, artesunate alone has little impact on cell numbers. Notably, mice receiving IMP, alone or in combination with artesunate, showed the largest reduction in circulating MV, which are associated with CM neuropathology and derived from monocytes and other cells in mice^39^ and human patients^38^. This reduction could be explained by a reduction in the numbers of cells from which they originate or by diminished activation of these cells^40,56^. The anti-TNF properties of artesunate alone evidently do not reduce circulating MV levels in infected mice, presumably because TNF is only one of many agonists present in severe malaria that can stimulate MV production^57^. In contrast, IMP treatment reduced the number of circulating cells, inevitably reducing MV numbers and may thus contribute to the efficacy of this therapy. Irrespective, the specific immune modulation of monocytes here shows significantly improved efficacy compared to current adjunctive general immune suppressive therapies.

In conclusion, through detailed analysis of the immune response in severe malaria, we identified the principal accumulating immune subsets and targeted them with a novel combination therapy. Synergy between IMP-mediated modulation of monocyte migration and the anti-parasitic/anti-inflammatory effects of artesunate in vivo resulted in almost complete protection against ECM and RD in mice with clinically evident severe malaria, as well as conferring robust long-term immunity. The late stage success of this combination therapy in mice highlights the novel potential of immune-modulatory treatments in severe malaria and suggests a potential avenue for human translation.

## Methods

### Mice

Female 7–12-week-old CBA/J mice were obtained from the Animal Resource Centre (ARC) (Western Australia, Australia) and kept under specific pathogen-free conditions with access to food and water ad libitum. Permission for all experiments was obtained from the University of Sydney Animal Ethics Committee and all experiments were performed in compliance with relevant University of Sydney, State and NH&MRC ethical regulations.

### *Plasmodium* infection

Experimental cerebral malaria (ECM) was induced by intraperitoneal injection of 1 × 10^6^ *Plasmodium berghei-*ANKA (PbA)-parasitised RBC (pRBC). pRBC were isolated from PbA-infected mice on d7 post infection (p.i.) and were stored in liquid nitrogen in Alsever’s solution with 10% glycerol until use^58^. Mock-infected mice received an i.p. injection of an equal volume of buffer. Mice were assessed using a previously described clinical evaluation score^59^ and diagnosed with ECM based on presentation with disease signs, including ruffled fur, severe motor impairment or convulsions (clinical score 3–4). Briefly, a score of 0 indicates absence of clinical signs; a score of 1 indicates non-ECM-specific signs such as ruffled fur and hunched posture; a score of 2 indicates some motor impairment and ruffled fur; a score of 3 indicates fitting and hemiplegia; and a score of 4 indicates coma. Parasitaemia was monitored by counting erythrocytes stained with Diff-Quick (ProSciTech, Queensland, Australia) on thin blood smears by light microscopy on d4 post infection (p.i.) and every 1–2 days after this, for the duration of the infection. Reinfection following resolution of primary infection was performed through administration of 1 × 10^6^ (PbA-pRBC) via intraperitoneal injection.

### Intravenous delivery of immune-modifying microparticles

Poly(lactic-co-glycolic acid) (PLGA) carboxylated IMP with a diameter of 500 nm were purchased from Phosphorex Inc (Hopkinton, MA, USA). IMP were diluted in sterile phosphate-buffered saline (PBS) to a concentration of 4.26 × 10^9^ particles/200 µL and administered intravenously.

### Administration of anti-malarial treatments

Quinine hydrochloride dihydrate, chloroquine diphosphate and artesunate (from *Artemisia annua*) were obtained from Sigma Aldrich (St. Louis, MO, USA). All anti-malaria drugs were diluted in sterile PBS to a concentration of 50 mg/kg in a 200 µL volume and administered intraperitoneally.

### Longitudinal studies

Longitudinal studies were performed to assess survival in treated, compared to non-treated mice. Mice treated from an early timepoint received IMP daily from d3–10 p.i., and three doses of quinine treatment from d14 p.i. Mice were treated when neurological ECM signs became evident and received IMP or artesunate, or a combination of IMP and either artesunate or chloroquine in two doses separated by 24 h.

### Cross-sectional studies

Cross-sectional studies were performed to assess the effect of treatment on histopathology and cellular infiltration at certain timepoints. This analysis took place at d8 or d10 p.i. in mice treated from d3 p.i. onwards, while mice treated from the onset of signs were sacrificed 36 h after initial treatment.

### Flow cytometry

Mice were anaesthetised, perfused with sterile PBS and the brain and lungs collected. The brains were processed into single cell suspensions as previously described^17^. Briefly, mechanical dissociation of the brain was followed by enzymatic digestion with DNase I (0.005 mg/mL) and collagenase (0.05 g/mL). Subsequently, a 30%/80%Percoll gradient was used to isolate the cells of interest from the digested tissue. Whole lungs were collected in 2 mL of RPMI with DNase I (0.2 mg/mL) and collagenase IV (0.2 mg/mL) (Sigma Aldrich) and the tissue cut up using C tubes on the gentleMACS dissociator (programme m_liver_03) (Miltenyi Biotec, Bergisch Gladbach, Germany) at room temperature. The samples were then incubated in a waterbath at 37 °C for 30 min, after which they were dissociated further on the gentleMACS (programme m_imp_04_01). Cell suspensions were strained through a 70 µm mesh cell strainer (Miltenyi Biotec) and red blood cells were lysed using RBC lysis buffer (Biolegend, San Diego, CA). Live cell numbers were determined by counting cells that excluded trypan blue. Single cell suspensions were incubated with anti-CD16/32 and LIVE/DEAD Fixable Blue Stain (Invitrogen, Carlsbad, CA) and subsequently stained with one of three cocktails of fluorescently labelled antibodies to identify myeloid, lung or brain populations. Fluorochrome-conjugated antibodies used were anti-CX3CR1 (SA011F11, Biolegend), anti-I-A/I-E (M5/114.15.2, Biolegend), anti-CD45 (30-F11, Biolegend), anti-NK1.1 (PK136, Biolegend), anti-Ly6G (1A8, Biolegend), anti-CD8α (53–6.7, Biolegend), anti-CD206 (C068C2, Biolegend), anti-CD11b (M1/70, Biolegend and BD Biosciences), anti-CD11c (N418, Biolegend and HL3, BD Biosciences), anti-Ly6C (HK1.4, Biolegend), anti-CD3ε (145-2C11, Biolegend and BD Biosciences), anti-B220 (RA3-6B2, Biolegend and BD Biosciences), anti-CD80 (16-01A1, Biolegend and BD Biosciences), anti-CD86 (GL-1, Biolegend), anti-CD4 (GK1.5, Biolegend), anti-CD64 (X54-5/7.1, Biolegend), anti-CD24 (M1/69, BD Biosciences), anti-Siglec-F (E50-2440, BD Biosciences), anti-CD115 (AFS98, Biolegend), anti-CCR2 (SA203G11, Biolegend), anti-F4/80 (BM8, Biolegend). Expression of cell surface markers was measured using the FACSDiva programme on a LSR II fluorescence-activated cell sorter (FACS) (Becton Dickinson, San Jose, CA). Acquired data was analysed in FlowJo (Tree Star Inc., Ashland, OR). Quality control gating based on forward and side scatter and LIVE/DEAD staining was applied to exclude debris, doublets and dead cells. Populations of interest were identified by fluorescence, according to the gating strategies shown in Supplementary Figs. 2 and 4, and quantified based on the percentage of the population and total cell counts.

### tSNE analysis

t-distributed stochastic neighbour embedding (tSNE) was applied to compensated populations of interest, exported from FlowJo. These files were loaded into Matlab R2014b using the cyt v3 tool, subsampled and transformed. Selected markers were used to perform a bh-tSNE analysis on the transformed data, resulting in a 2D plot, which was overlaid with a heat map of marker expression^22^. The markers used for the generation of brain tNSE plots were CD11b, CD45, CD11c, CX3CR1, CCR2, Ly6G, F4/80, MHC-II, CD80, CD86, CD205 and CD206 with a perplexity parameter of 30. The markers used for the generation of lung tSNE plots were CD11b, CD45, CD11c, CD3e, CD24, CD64, CD80, CD86 and CD206 with a perplexity parameter of 30. A plot showing expression of each marker was generated using a script in R^60^. MFI of brain and lung populations used in tSNE analysis is shown in Supplementary Tables 1 and 2, respectively.

### Histology

Brain, lung and spleen tissue was fixed in neutral-buffered formalin for 24 h, and 70% ethanol for 48 h. Tissue was then dehydrated overnight, embedded in paraffin wax, cut into 5 µm thick sections, mounted on slides and stained using standard haemotoxylin and eosin protocols. Histopathology was assessed using a previously defined scoring system^61^. The proportion of occluded vessels (containing leucocytes and/or RBC) to empty vessels was determined on blinded sections from each of the treatment and control groups, as previously described^62^. No specific area of the brain was chosen for this examination, as this is typically a diffuse pathology, with instances of sequestration and haemorrhaging observed throughout the brain. Thus, the entire brain section was examined in each case.

### Cell sorting and adoptive transfer

The bone marrow of d7 p.i. PbA-infected mice was isolated from femurs and tibias and processed into a single cell suspension. Two donors were used per recipient. Red blood cells were lysed using RBC lysis buffer (Biolegend, San Diego, CA). Subsequently, cells were incubated with anti-CD16/32 and LIVE/DEAD Fixable Blue Stain (Invitrogen, Carlsbad, CA). Next, the cells were incubated with fluorescently-labelled antibodies against CD45, CD115, CD11b, Ly6C, Ly6G and B220. Ly6C^hi^ CD11b^+^ inflammatory monocytes were sorted on an Influx cell sorter using the FACSDiva Programme (Becton Dickinson). The gating strategy is shown in Supplementary Fig. 3. The purity of the sorted cells was > 90% in all cases and 5 × 10^5^ cells were transferred to each recipient. These cells were labelled with membrane dye PKH26 (Invitrogen) according to the manufacturers’ instructions and injected intravenously into PbA-infected recipients in 200 µL sterile PBS on d7 p.i. Recipients were sacrificed 12, 24 and 36 h post-transfer and the brain and lungs were isolated and processed for flow cytometry, as described above.

### Microvesicle (MV) enumeration

Mouse venous blood was collected by retro-orbital puncture under anaesthesia in 0.129 mol/L sodium citrate (ratio of blood to anticoagulant 4:1). Samples were centrifuged at 1500x*g* for 15 min at room temperature. Harvested, platelet-poor plasma samples were further centrifuged at 18,000x*g* for 5 min, twice, to achieve platelet-free plasma. Platelet-free plasma was centrifuged at 18,000x*g* for 45 min, the supernatant removed and retained, and the pellet centrifuged for a further 45 min at 18,000x*g* in a solution of sodium citrate and PBS (ratio of citrate to PBS 1:3) to pellet MV. Total MV numbers were quantified by detection of phosphatidylserine using FITC-Annexin V labelling (Beckman Coulter, Brea, CA), as previously described^41^. Samples were acquired on a Gallios flow cytometer (Beckman Coulter) and analysed using Kaluza (Beckman Coulter).

### Statistical analysis

GraphPad Prism 7 (GraphPad Software, La Jolla, CA) was used to graph data and perform statistical analyses. Survival data was compared using the Mantel-Cox log-rank test. Comparison of two samples was conducted using Mann–Whitney, and three or more samples were compared using Kruskal–Wallis test with a Dunn’s multiple comparison test. *p*-values of  ≤0.05 were regarded as significant and designated in figures as *p* ≤ 0.05 (*), *p* ≤ 0.01 (**), *p* ≤ 0.005 (***) and *p* ≤ 0.001 (****). Error bars are shown as standard error of the mean (SEM).

### Code availability

The custom code used in R for coloration of tSNE plots is publicly available^60^.

## Electronic supplementary material

Supplementary Information

Source Data

## Data Availability

The data that support the findings in this study are available from the corresponding author upon reasonable request. The source data of each figure are presented as a Source Data file.
