# Supplementary material for: Experimental severe malaria is resolved by targeting newly-identified monocyte subsets using immune-modifying particles combined with artesunate
Source: Commun Biol. 2018 Dec 13;1:227. doi: 10.1038/s42003-018-0216-2 (PMC6292940; doi:10.1038/s42003-018-0216-2)
Supplement: Supplementary file 1 — Supplementary Information [file 42003_2018_216_MOESM1_ESM.pdf]

Supplementary figure 1

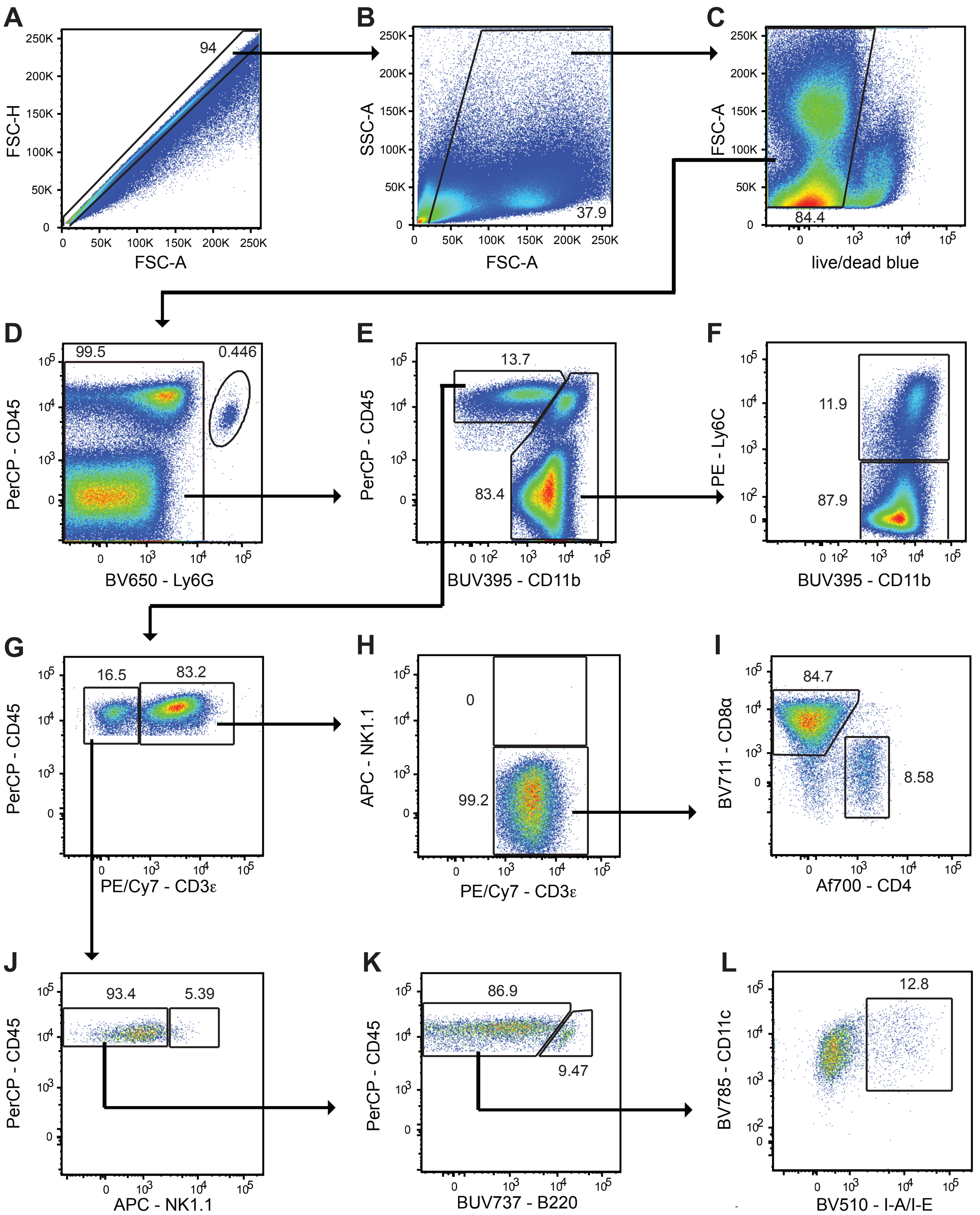

Supplementary Figure 1. Gating strategy for brain populations
Representative sequential flow cytometric gating used to identify cell populations in the PbA-infected brain on d8 p.i. Doublets (**A**), debris (**B**) and dead cells (**C**) were excluded. Neutrophils were gated as Ly6G$^+$ CD45$^+$ cells (**D**) and the remaining population was further divided into CD11b$^+$ myeloid and CD45^{hi} lymphocytes (**E**). Myeloid cells were split into Ly6C^{lo} and Ly6C^{hi} cells (**F**). CD3ε$^+$ cells (**G**) were split into CD3ε$^+$ NK1.1$^+$ NKT cells and CD3ε$^+$ NK1.1$^-$ T cells (**H**). T cells were further characterised as CD4$^+$ or CD8$^+$ T cells (**I**). CD3ε$^-$ cells were identified as NK1.1$^+$ NK cells (**J**), B220$^+$ B cells (**K**) and CD11c$^+$ MHC-II$^+$ classical DC (**L**).

Supplementary figure 2

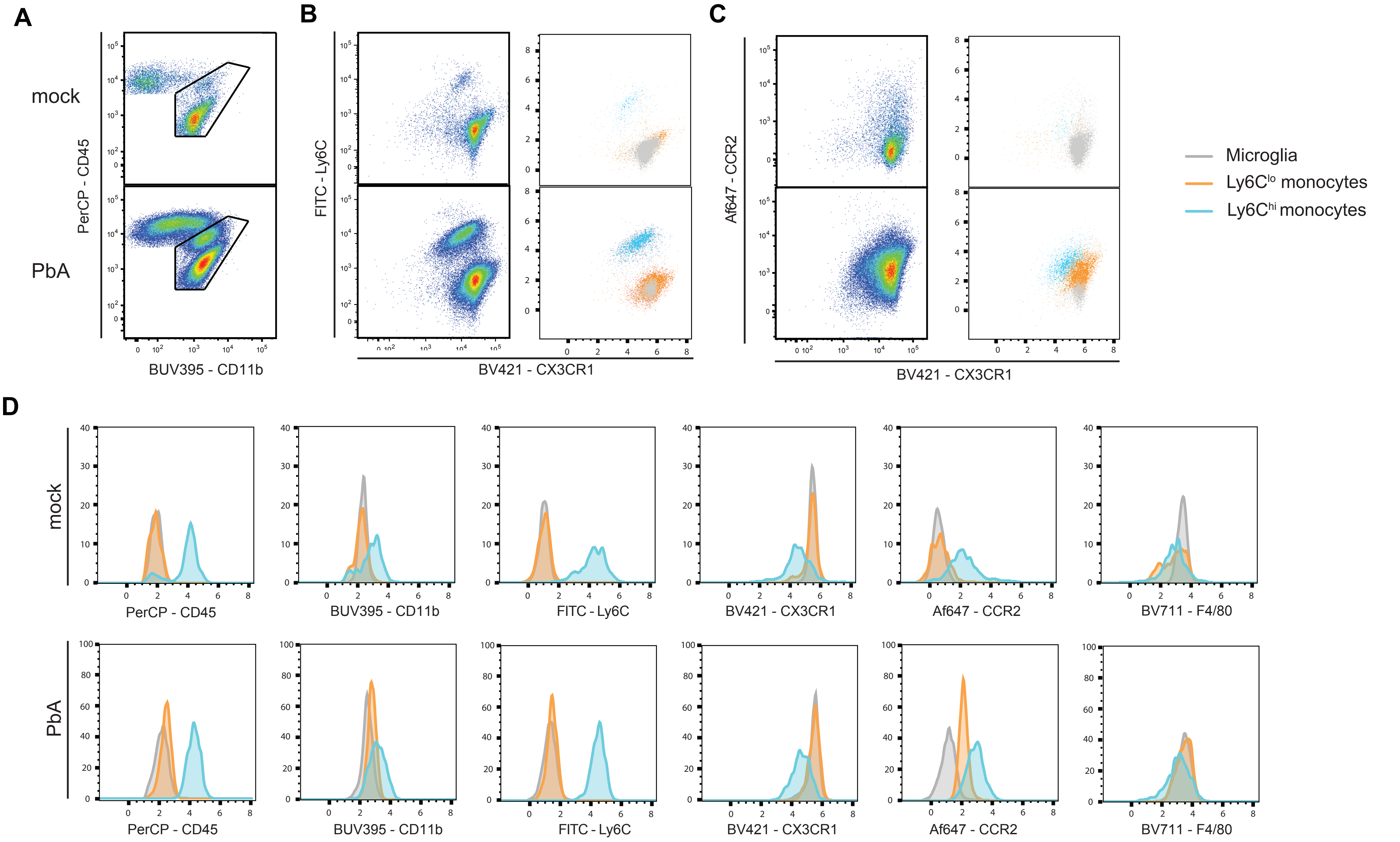

Supplementary figure 2. Detailed analysis of CD11b+ brain population
A Gating of the CD11b+ population in the brain of mock- and d8 p.i. PbA-infected mice. **B-C** Dot plots of the $CD11b^+$ population plotted as Ly6C vs CX3CR1 (**B**) and CCR2 vs CX3CR1 (**C**) by manual analysis in FlowJo (left panels) and by overlaying the three populations from tSNE analysis onto the same plot (right panels). **D** Representative histograms showing expression of markers on the three myeloid populations resolved by tSNE in the brains of mock- and PbA-infected mice.

Supplementary figure 3

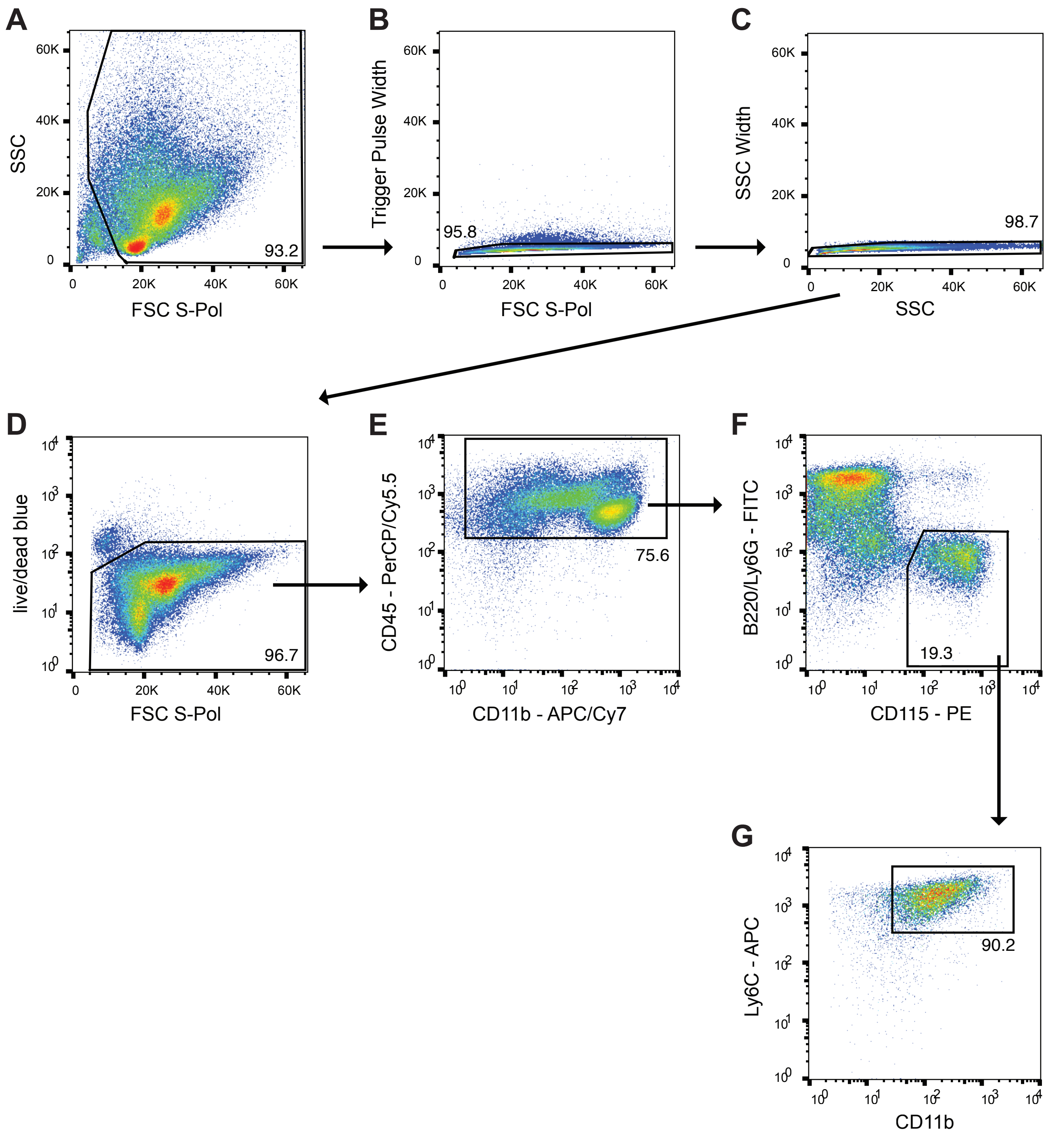

Supplementary figure 3. Gating strategy for sorting Ly6C^{hi} monocytes from bone marrow isolates
Representative sequential flow cytometric gating used to sort Ly6C^{hi} monocytes from the bone marrow. Exclusion of debris (**A**), doublets (**B** and **C**) and dead cells (**D**) was applied. Subsequently CD45^{+} leukocytes were gated (**E**) and B220^{-}Ly6G^{-}CD115^{+} monocytes (**F**) were selected. Of these, CD11b^{+} monocytes with high expression of Ly6C were sorted (**G**).

Supplementary figure 4

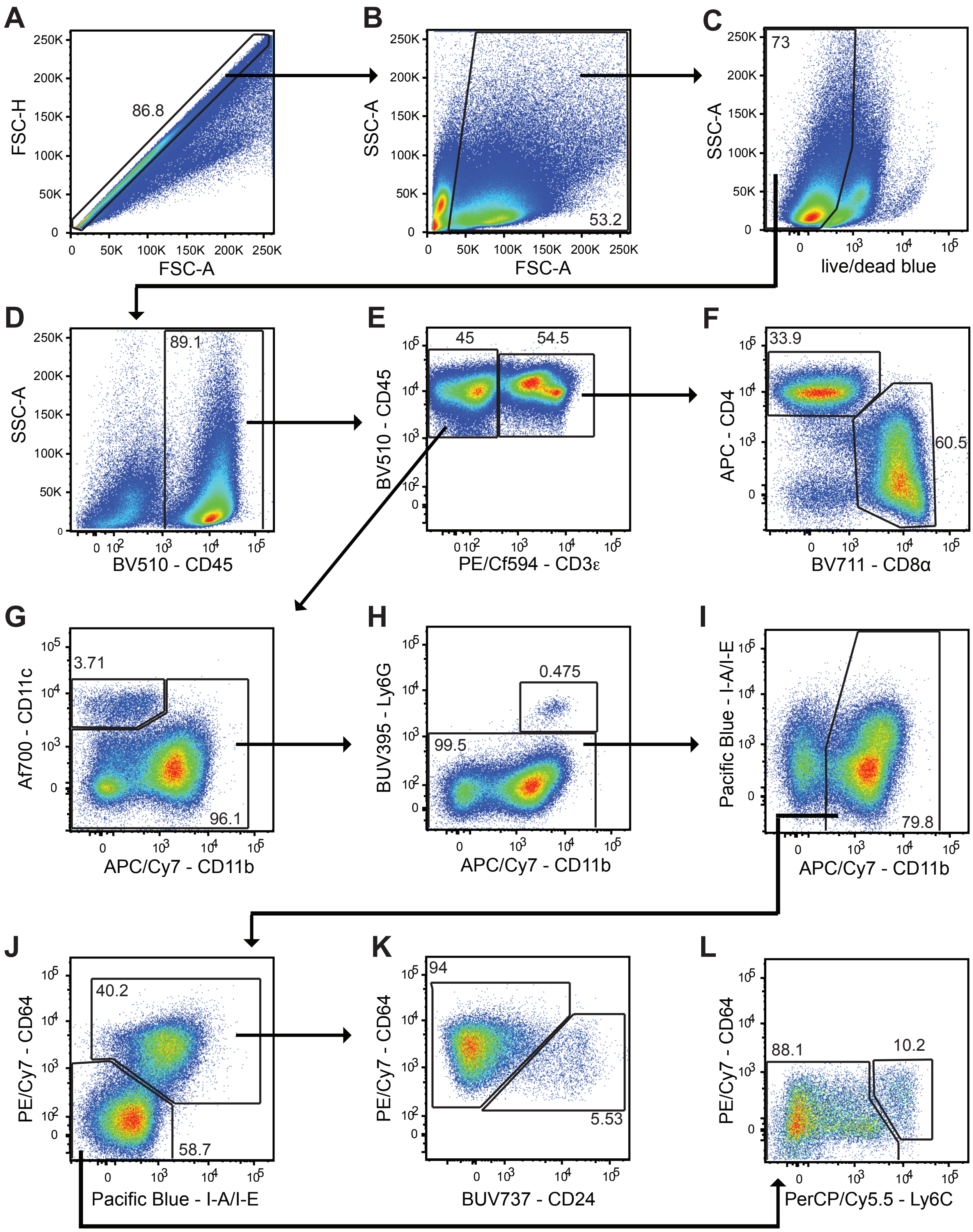

Supplementary figure 4. Gating strategy of the lung
Representative sequential flow cytometric gating used identify cell populations in the PbA-infected lung on d8 p.i. Doublets (**A**), debris (**B**) and dead cells (**C**) were excluded. CD45 expression was used to gate the total leukocyte population (**D**). T cells were identified as $CD3\varepsilon^+$(**E**) and further characterised as $CD4^+$ or $CD8^+$ (**F**). In the $CD3\varepsilon^-$ population, alveolar macrophages were identified as $CD11c^+$ $CD11b^{lo}$ (**G**). Neutrophils were gated as $CD11b^+$ $Ly6G^+$ (**H**). The remaining $CD11b^+$ population (**I**) was split into $CD64^-$ MHC-II^- cells and $CD64^+$/MHC-II^+ (**J**). Subsequently, CD64 and CD24 expression of $CD64^+$/MHC-II^+ cells was used to identify $CD24^-$ interstitial macrophages and $CD24^+$ $CD11b^+$ DC (**K**). $CD64^-$ MHC-II^- cells were divided into $Ly6C^{lo}$ and $Ly6C^{hi}$ monocytes (**L**).

Supplementary figure 5

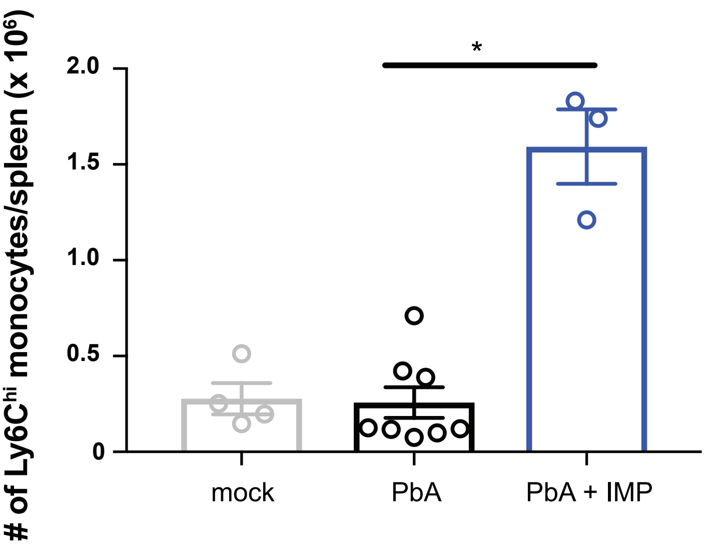

Supplementary figure 5. Ly6C^{hi} monocytes accumulate in the spleen of IMP-treated mice
The number of Ly6C^{hi} monocytes in the spleen of mock-infected, PbA-infected and PbA-infected, IMP-treated mice on d8 p.i. Data represents 1 experiment with a total n of 3-8 mice per group, shown as mean ± SEM and analysed using a Kruskal-Wallis test with a Dunn's multiple comparison test.

Supplementary Figure 6

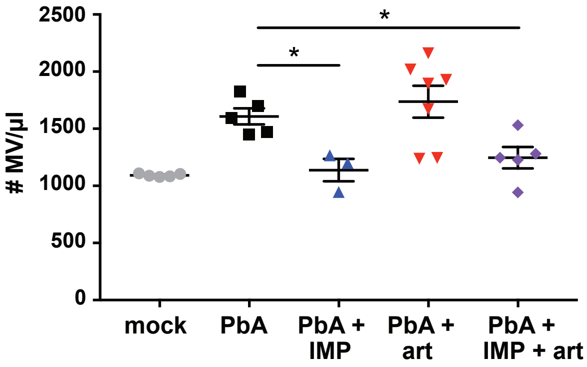

Supplementary figure 6. Effect of treatment on circulating microvesicles
Number of circulating microvesicles per microlitre of platelet-free plasma in mock-infected mice, and in PbA-infected mice, comparing IMP, artesunate or combination treatment from d7 p.i. with untreated controls. Data represents 1 experiment with a total n of 3-7 mice per group shown as mean ± SEM and were analysed using a Mann-Whitney test.

Supplementary Figure 7

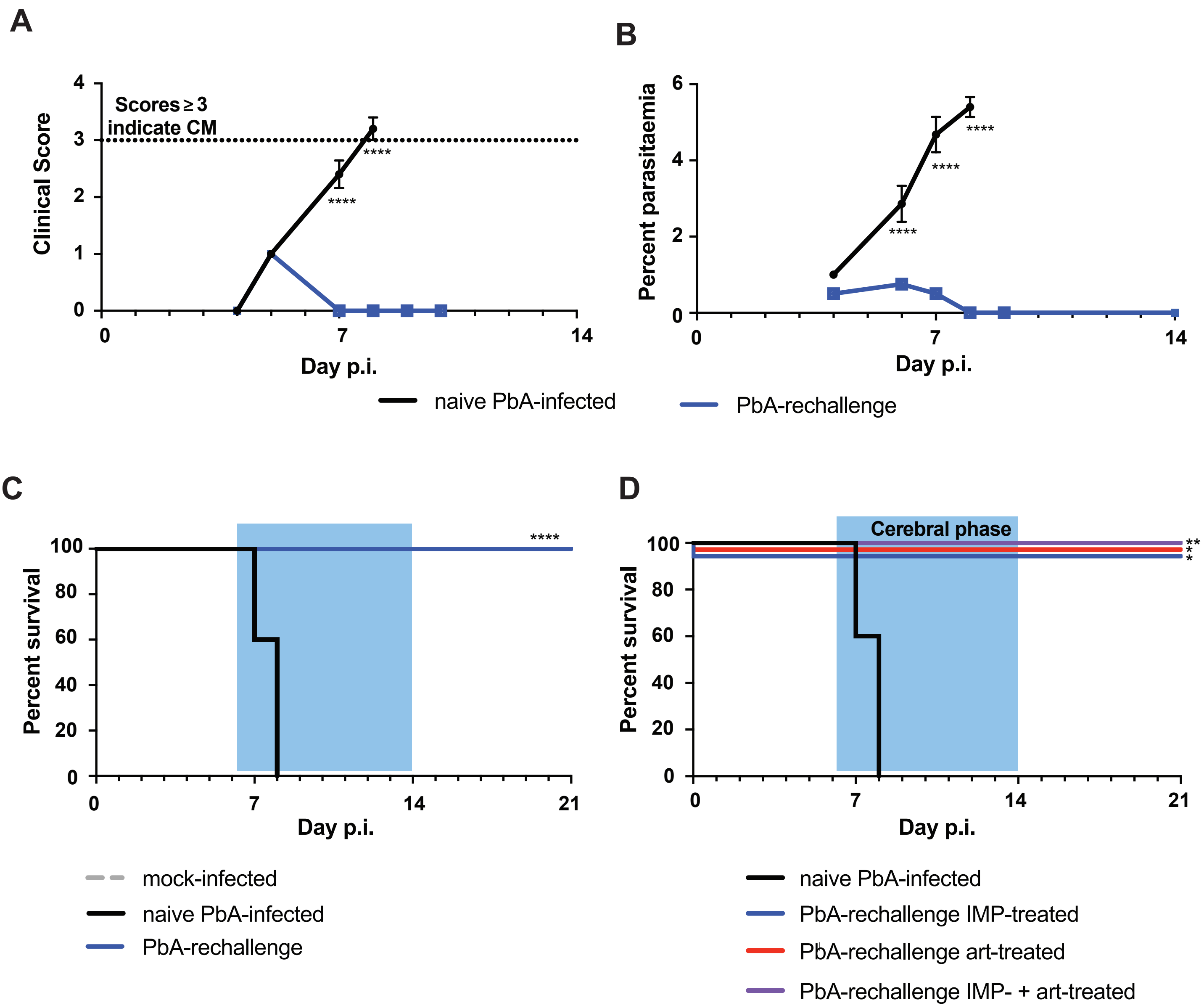

Supplementary figure 7. Treated PbA-infected mice are protected during reinfection
A-B Clinical scores (**A**) and parasitaemia (**B**) in previously successfully treated mice, reinfected on d35 p.i., compared to naïve PbA-infected mice. **C** Survival of mice reinfected with PbA, following previous successful early IMP treatment, compared with untreated controls. The acute phase of mortality, observed during the second week post-PbA-infection, is denoted by the blue box. **D** Survival of mice reinfected with PbA, following previous successful late IMP, artesunate or combination treatment, compared with untreated controls. All rechallenged groups had 100% survival, but the lines have been separated for clarity. The acute phase of mortality, observed during the second week post-PbA-infection, is denoted by the blue box. Data in figures A-C represents 3 separate experiments with a total n of 5-16 mice per group and data in figure D represents 2 separate experiments with a total n of 3-6 mice per group. Data in figures A-B are shown as mean ± SEM and analysed using a Kruskal-Wallis test with a Dunn's multiple comparison test comparing all groups at each timepoint. Data in figure C and D were compared using the Mantel-Cox log-rank test.

		Microglia	Ly6C^{lo} monocytes	Ly6C^{hi} monocytes
**CD45**	mock	2.032 ± 0.015	2.815 ± 0.034	3.848 ± 0.115
	PbA	2.050 ± 0.055	2.775 ± 0.076	4.463 ± 0.090
**Ly6C**	mock	1.320 ± 0.028	1.718 ± 0.057	3.993 ± 0.051
	PbA	1.243 ± 0.034	1.628 ± 0.062	4.348 ± 0.045
**F4/80**	mock	3.328 ± 0.050	4.043 ± 0.061	2.753 ± 0.107
	PbA	2.848 ± 0.200	3.518 ± 0.105	3.053 ± 0.230
**CD80**	mock	1.678 ± 0.009	2.355 ± 0.069	1.563 ± 0.123
	PbA	1.595 ± 0.033	2.510 ± 0.082	3.058 ± 0.288
**MHC-II**	mock	2.923 ± 0.048	4.018 ± 0.075	3.663 ± 0.135
	PbA	3.080 ± 0.042	3.920 ± 0.042	4.648 ± 0.220
**CX3CR1**	mock	5.480 ± 0.064	5.553 ± 0.094	4.495 ± 0.103
	PbA	5.043 ± 0.202	5.483 ± 0.074	4.448 ± 0.122
**CD86**	mock	1.005 ± 0.006	1.180 ± 0.018	1.045 ± 0.013
	PbA	1.005 ± 0.006	1.088 ± 0.017	1.068 ± 0.013
**CCR2**	mock	1.080 ± 0.021	2.480 ± 0.076	2.076 ± 0.015
	PbA	1.278 ± 0.050	2.505 ± 0.095	2.960 ± 0.158

Supplementary table 1. MFI of brain populations in tSNE analysis

MFI of indicated markers on populations in the brain of mock- and PbA-infected mice on d8 p.i., as determined by tSNE analysis. Fluorescent markers are scaled during tSNE analysis and range from 0-6.

		Alveolar macrophages	Interstitial macrophages	Ly6C^{lo} monocytes
**CCR2**	mock	3.477 ± 0.055	3.737 ± 0.006	2.590 ± 0.044
	PbA	4.565 ± 0.304	4.965 ± 0.007	2.800 ± 0.057
**CD86**	mock	2.817 ± 0.055	2.683 ± 0.108	2.300 ± 0.010
	PbA	2.970 ± 0.001	2.745 ± 0.021	2.240 ± 0.001
**CD11b**	mock	3.433 ± 0.035	6.000 ± 0.096	5.877 ± 0.081
	PbA	4.220 ± 0.2545	6.000 ± 0.064	5.355 ± 0.163
**CX3CR1**	mock	3.767 ± 0.032	4.533 ± 0.091	2.853 ± 0.072
	PbA	4.330 ± 0.3677	4.770 ± 0.014	3.370 ± 0.255
**MHC-II**	mock	5.350 ± 0.030	5.720 ± 0.125	3.170 ± 0.060
	PbA	5.000 ± 0.056	5.635 ± 0.106	2.665 ± 0.049
**CD80**	mock	4.766 ± 0.091	2.49 ± 0.1769	1.191 ± 0.099
	PbA	3.750 ± 0.665	4.125 ± 0.007	1.905 ± 0.050
**CD206**	mock	5.086 ± 0.070	3.080 ± 0.265	1.923 ± 0.006
	PbA	2.820 ± 0.679	1.650 ± 0.184	2.070 ± 0.042
**Ly6C**	mock	4.573 ± 0.035	4.240 ± 0.261	3.110 ± 0.115
	PbA	4.235 ± 0.304	5.945 ± 0.007	3.060 ± 0.071
**CD64**	mock	5.333 ± 0.049	4.733 ± 0.072	2.327 ± 0.023
	PbA	5.120 ± 0.537	5.650 ± 0.226	2.385 ± 0.064

Supplementary table 2. MFI of lung populations in tSNE analysis
MFI of indicated markers on populations in the lung of mock- and PbA-infected mice on d8 p.i., as determined by tSNE analysis. Fluorescent markers are scaled during tSNE analysis and range from 0-6.
